# Prevalence and Correlates of Anxiety in Fort McMurray Vulnerable Population during the COVID-19 Pandemic

**DOI:** 10.1192/j.eurpsy.2023.970

**Published:** 2023-07-19

**Authors:** R. Shalaby, E. Eboreime, N. Nkire, B. Agyapong, H. Pazderka, G. Obuobi-Donkor, M. Adu, W. Mao, E. Owusu, F. Oluwasina, V. Agyapong

**Affiliations:** 1 Department of Psychiatry, University of Alberta, Edmonton; 2 Department of Psychiatry, Dalhousie University, Halifax, Canada

## Abstract

**Introduction:**

The COVID-19 pandemic has produced negative mental health outcomes, which were more prominent in vulnerable communities, such as Fort McMurray (FMM), the community that experienced prior similar disasters.

**Objectives:**

This study aimed to examine the likelihood and correlates of anxiety symptoms among FMM residents, during the COVID-19 pandemic.

**Methods:**

A cross-sectional online survey questionnaire was applied between 24 April and 2 June 2021, at FMM community to gather a set of data, including sociodemographic, COVID-19, and clinical information. Generalized anxiety disorder was the main outcome of the study, and was measured using GAD-7 scale.

**Results:**

Overall, 186 individuals completed the survey (response rate 74.7%). Most of the respondents were females (159, 85.5%); above 40 years (98, 52.7%); employed (175, 94.1%); and in relationship (132, 71%). The prevalence of moderate-to-severe anxiety was (42.5%, 71) on GAD-7 self-reported scale. Subscribers who reported that they would like to receive mental health support; have received no family support since COVID-19 declaration; and have lost their job during the pandemic were all more likely to report moderate-to-severe anxiety (OR = 3.39; 95% CI: 1.29-8.88), (OR = 4.85; 95% CI: 1.56-15.03), and (OR = 4.40; 95% CI: 1.01-19.24), respectively.

**Conclusions:**

Anxiety levels were high among FMM residents, compared to levels before COVID-19. Clinical and social factors related to the COVID-19 pandemic significantly predicted likely anxiety among Fort McMurray population. It is imperative to mobilize resources to support vulnerable communities during the COVID-19 pandemic.

**Disclosure of Interest:**

None Declared

